# Achieving ‘Marginal Gains’ to Optimise Outcomes in Resectable Pancreatic Cancer

**DOI:** 10.3390/cancers13071669

**Published:** 2021-04-01

**Authors:** Sarah Powell-Brett, Rupaly Pande, Keith J. Roberts

**Affiliations:** 1 Department of Hepatopancreatobiliary Surgery and Liver Transplantation, University Hospitals Birmingham NHS Foundation Trust, Birmingham B15 2GW, UK; rpande@nhs.net (R.P.); Keith.Roberts@uhb.nhs.uk (K.J.R.); 2 Institute of Immunology and Immunotherapy, University of Birmingham, Birmingham B15 2TT, UK

**Keywords:** pancreatic cancer, pancreatic exocrine insufficiency, adjuvant chemotherapy, biliary drainage, prehabilitation, ERAS

## Abstract

**Simple Summary:**

Improving outcomes in pancreatic cancer is achievable through the accumulation of marginal gains. There exists evidence of variation and undertreatment in many areas of the care pathway. By fully realising the existing opportunities, there is the potential for immediate improvements in outcomes and quality of life.

**Abstract:**

Improving outcomes among patients with resectable pancreatic cancer is one of the greatest challenges of modern medicine. Major improvements in survival will result from the development of novel therapies. However, optimising existing pathways, so that patients realise benefits of already proven treatments, presents a clear opportunity to improve outcomes in the short term. This narrative review will focus on treatments and interventions where there is a clear evidence base to improve outcomes in pancreatic cancer, and where there is also evidence of variation and under-treatment. Avoidance of preoperative biliary drainage, treatment of pancreatic exocrine insufficiency, prehabiliation and enhanced recovery after surgery, reducing perioperative complications, optimising opportunities for elderly patients to receive therapy, optimising adjuvant chemotherapy and regular surveillance after surgery are some of the strategies discussed. Each treatment or pathway change represents an opportunity for marginal gain. Accumulation of marginal gains can result in considerable benefit to patients. Given that these interventions already have evidence base, they can be realised quickly and economically.

## 1. Introduction

Pancreatic cancer is projected to become the second leading cause of all cancer-related deaths by 2030 [1,2]. Although surgical resection is the foundation of ‘resectable’ pancreatic cancer management, alone it is associated with a less than 10% chance of cure [3]. High rates of perioperative morbidity and mortality, slow or poorly organised pathways to surgery, suboptimal preoperative management of jaundice, suboptimal use of (neo)adjuvant therapy and failure to address malnutrition all contribute to poor outcomes. It is therefore unsurprising that there can be a negative attitude and feelings of nihilism when considering the outlook for these patients.

Wide variations in care and a lack of standardised practice, however, offer an easy way to improve outcomes in the near future. Pathways to expedite surgery, prescribing of enzyme therapy, access to adjuvant chemotherapy and tackling frailty are just a few examples of how care has the potential to be optimised. Optimising an individual aspect of care represents an opportunity for a marginal gain. Individually, each gain realised may be relatively minor when observed across an entire patient cohort, but when multiple, the effect of aggregated marginal gains can be considerable. The aggregation of marginal gains was popularised in elite level cycling where its success was clear to see at Olympic level and major events such as the Tour de France. The principals have been adopted in healthcare among various patient populations from cancer surgery, stroke recovery, prehabilitation, cardiac surgery and anaesthesia [4,5,6]. This narrative review highlights common failings in the care of pancreatic cancer patients and describes where gains can be made. The aggregation of these marginal gains could improve outcomes and experience for a great many patients with resectable pancreatic cancer.

## 2. Pre-Operative Pathways

At presentation, resectable pancreatic cancer is in an exponential phase of growth and most primary tumours harbour cells that can metastasise [7]. Enhancing pre-operative pathways to ensure that patients are treated as quickly as possible and their functional status is optimised is essential to enabling the delivery of the highest standard of care. This section will focus on avoidance of pre-operative biliary drainage, prehabilitation, and optimisation of nutritional status.

### 2.1. Preoperative Biliary Drainage in Resectable Pancreatic Cancer

The majority of patients with resectable pancreatic cancer present with jaundice. Historically reluctance to operate in the presence of jaundice was related to concerns over renal, cardiac and liver dysfunction, coagulopathy and, at the time, high rates of perioperative morbidity and mortality associated with pancreatic surgery in the absence of jaundice [8].

Hence, in theory, relief of jaundice by preoperative biliary drainage (PBD) is perceived to improve these disturbances and prevent postoperative complications, though in practice, the role and propriety of PBD must be challenged.

The concept of correcting jaundice prior to resection was introduced by A.O. Whipple through staged PD, initially a cholecystogastrostomy to relieve jaundice followed by resection once the jaundice was within ‘safe’ limits. This further developed into nonoperative approaches through percutaneous transhepatic cholangiopathy (PTC) and biliary drainage in the 1960s and later followed by endoscopic retrograde cholangiopancreatography (ERCP) in the 1970s [9,10].

For many decades ERCP served both diagnostic and therapeutic purposes. However, the sensitivity and specificity of CT to diagnose pancreatic cancer has made ERCP, as a diagnostic tool, obsolete.

The main drawback of PBD is the associated rate of complications. These complications impair quality of life, can delay or prevent future surgery and are occasionally fatal. PBD itself requires resource and invariably delays treatment pathways [11,12].

The DROP trial randomised patients to upfront early surgery within 7 days or PBD-and plastic stent and delayed surgery between 4–6 weeks [13]. This landmark RCT showed the rate of serious complications at 120 days postoperatively to be far higher in the PBD group (74 vs. 39%, *p* < 0.001). This was largely due to drainage-related complications, cholangitis (26 vs. 2%), pancreatitis (7 vs. 0%) along with the need for change of stent in 30% of patients. There are also higher rates of perioperative infections, associated with changes in the biliary microbiome related to PBD [14,15] It is therefore advised that intraoperative bile cultures are taken and appropriate antibiotic administered to address this higher rate of complications [16].

Self-expanding metal stents (SEMS) have gained in popularity over plastic stents based on significantly greater patency and reduction in infectious complications [13,17,18]. The experience of patients undergoing PBD with fully covered SEMS, with a prospective study which mirrored the protocol of the DROP trial, superimposed the complication rates of PBD-SEMS over the cohorts within the DROP trial [19]. Though SEMS were associated with lower rates of complications than plastic stents, the rate remained significantly greater than upfront surgery (51 vs. 39%). Subsequent meta-analysis confirms advantages of metal over plastic stents with reduced rates of need for endoscopic reintervention (OR0.3), preoperative complications (OR 0.42) and cholangitis (OR 0.09) [20]. However, pancreatitis is more common (OR 3.6) and, though not reported, post hoc analysis of resection rates demonstrates a lower resection rate among patients with metal stents (plastic, 147/190 vs. metal 93/139; Chi square *p* = 0.04) [19,20]. A more recent network meta-analysis demonstrated a remarkably high rate of complications with plastic stents (38–93%), a much lower rate with SEMS (0–15%) whilst data regarding outcomes from percutaneous drainage is not at extensively reported (31%) [21]. This study also concluded that avoidance of PBD was associated with the best outcome.

The association between PBD and reduced resection rates was not observed in individual randomised trials. However, it is logical to associate complications such as pancreatitis or pathway delays with a reduction in resection rate. Many high-volume centres have reported that increasing time to surgery reduces resection rates, data confirmed within a recent systematic review and meta-analysis [22,23,24,25]. It may be that this higher resection rate and avoidance of complications translates into increased survival benefit, when analysed on an intention to treat basis [26]. This is controversial as some authors have associated hyperbilirbuinaemia at the time of resection with worse cancer outcomes [27].

Given the many benefits of avoiding PBD, the National Institute for Health and Care Excellence (NICE) in the United Kingdom, now recommend upfront surgery without PBD where possible [28].

What remains to be defined is a safe upper limit of bilirubin in terms of safety and oncologic outcomes and why PBD remains so widely used despite the evidence of harm. Associated venous resection appears safe in the presence of jaundice, even when levels of bilirubin exceed 300 µmol/L (>17.5 mg/dL) [29]. It may be assumed that elderly patients are safer to undergo PBD and surgery rather than upfront surgery but elderly patients are less likely to tolerate complications of PBD and remain on a surgical pathway than younger patients.

Numerous studies have established seemingly arbitrary upper limits of bilirubin at which PBD is indicated [27,30,31,32,33,34,35]. A common threshold of >250 umol/L is used and cited by The Guidelines for Perioperative Care for Pancreaticoduodenectomy: Enhanced Recovery After Surgery (ERAS^®^) Society Recommendations [36]; Both Sauvanet and Li et al. reported higher complication rates when surgery was performed with a bilirubin of >300 umol/L [27,37]. However, contrary to this, van der Gaag, quoted a bilirubin level of ≤300 μmol/L at surgery and demonstrated significantly lower infective complications, whilst Pamecha et al. showed that a bilirubin level of ≥15 mg/dL (≥265 μmol/L) was not an independent risk factor for complications [13,38,39].

As stated above, it is the authors experience that surgery, even with venous resection, can be performed with no difference in complications with bilirubin in excess of 300 μmol/L. To summarise, there is no clear data that defines an upper limit of bilirubin at which PBD is indicated. The authors recommend an approach with prospective evaluation of outcomes and a step wise increase in threshold of bilirubin at which to undertake PBD. Variation in the rate of increase of bilirubin, the difficulty/time needed to complete staging tests and other factors such as renal function or evidence of biliary sepsis mean that there is unlikely to be a single value which can be applied to all patients.

It is useful to consider when PBD is indicated. Jaundiced patients undergoing neoadjuvant therapy clearly require PBD; if there are major diagnostic delays, though an assessment of risk of cancer progression must be weighed up against potential risks of surgery in such cases or if the patient is too frail to undergo surgery. Such patients, however, in our experience rarely improve significantly after PBD. Cholangitis has been considered a contraindication for PBD, though in our practice we frequently employ external biliary drainage, antibiotics, fluid replacement therapy and surgery within the same week if markers of infection are improving. This has controlled sepsis, avoided complications of PBD and kept patients within an early surgery pathway. See Table 1. For a summary of potential indications for PBD and Table 2 for optimizing care should PBD be undertaken.

Malnutrition associated with obstructive jaundice and its effects on outcomes following surgery is a further area of controversy. In studies by Padillo et al., malnutrition was more commonly associated with patients older than 68 years and those with high levels of bilirubin with the suggestion that PBD should be considered to allow alleviation of these modifiable factors preoperatively [40,41]. However, evidence for the optimal duration of PBD to improve nutritional status is experimental and its role in malnutrition must be considered in the broader context of pancreatic exocrine insufficiency [42,43,44,45]. Exocrine insufficiency is prevalent at diagnosis, and although not the sole cause of malnutrition, it is a major driver of malnutrition and is poorly treated; thus, delays to surgery can exacerbate malnutrition if PEI is not addressed. A balance must be struck between early surgery and better treatment of PEI versus correcting jaundice and improving nutrition. A proposed optimal pathway would be to provide PERT for all patients and provide early surgery where possible; where a patient has a poor performance status with malnutrition, a pathway of PBD, PERT, and dietician input would be advocated.

### 2.2. Pancreatic Exocrine Insufficiency and Overcoming Malnutrition to Improve Outcomes

Pancreatic exocrine insufficiency (PEI) and pancreatic enzyme replacement therapy (PERT) should be considered at all stages of management of pancreatic cancer, it is included in this section on pre-operative pathways as correct treatment is essential as early in the care pathway as possible. PEI is far from trivial and should be considered as organ failure. Untreated failure of other organ systems can be rapidly fatal (for example, renal, cardiac or respiratory failure or even diabetes) and withholding treatment in these settings is reserved only for those patients who are on end-of-life pathways. Consequently, it is remarkable that PEI is underdiagnosed and undertreated. *In the authors view, this single issue represents the simplest pathway improvement in this review and also the intervention with the chance to achieve most gain.*

**Figure 1:**
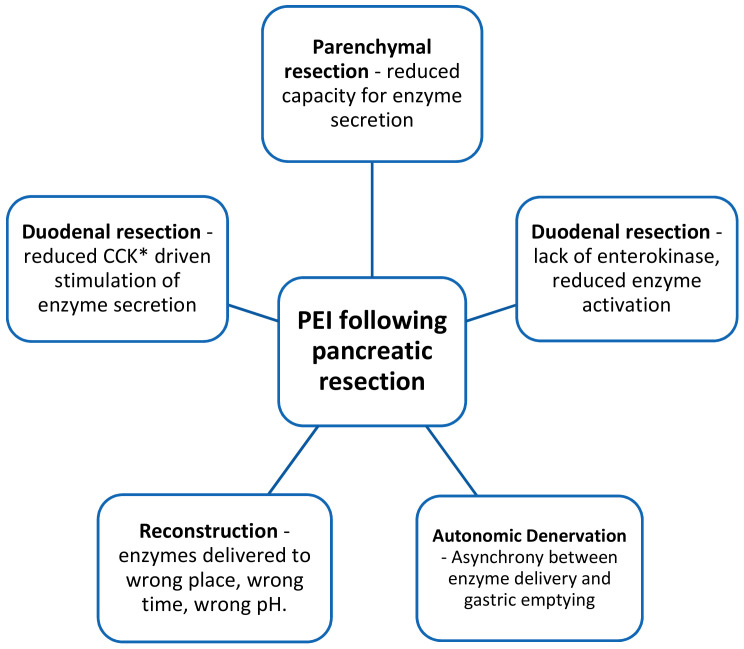
Factors contributing to PEI following pancreatico-duodenectomy. *CCK = cholecystokinin.

Addressing pancreatic exocrine insufficiency (PEI) can have an effect upon survival as strong as that as surgery or chemotherapy and yet many patients remains untreated [46]. A 2016 systematic review of PEI demonstrated a pre-operative prevalence of 44% and a post-operative prevalence of 74% (for those undergoing pancreatico-duodenectomy for malignancy). This is likely a conservative estimate owing to the frequent use of faecal elastase (FE-1) to diagnose PEI (FE-1 has been shown to underestimate PEI following resection) [47,48]. Furthermore, there is a gradual reduction of exocrine function at a median value of 10% per month [49]. With the gold standard of testing and longer term follow up, Lemaire et al. found the post-operative incidence of PEI to be 94% [50]. In resectable disease the mechanisms underlying PEI are complex and multifactorial. Almost all physiologic control is lost after pancreatoduodenectomy, and together with other factors result in insufficient enzymes arriving at the wrong time, to the wrong place and at the wrong pH for effective function [51,52] (see Figure 1).

Symptoms of PEI include pain, bloating, frequency, urgency, diarrhoea, fatty stool, flatulence, loss of appetite, nausea and vomiting and are physically and mentally distressing. The ‘classical’ sign of steathorrhoea is often absent, either because PEI is not severe enough or because the patient may have unconsciously adopted behaviours to avoid fat intake [53,54,55]. PEI is frequently untreated or undertreated worldwide. Studies from the UK, Europe and Australia demonstrate that only a minority of patients receive PERT [56,57,58,59]. Reasons for under prescribing are multifactorial and may relate to the lack of an acceptable or accurate routine diagnostic test that can yield results in near real time [47,60,61,62]. Confusing symptoms of cancer, weight loss and abdominal discomfort, further complicate diagnosis.

The consequences of PEI must not be underestimated, these include: weight loss, malnutrition, micronutrient deficiency, cardiovascular events, osteoporosis, fractures and sarcopenia [63]. The effect of PEI on operative outcomes is considerable, being associated with higher rates of post-operative complications, longer hospital stays and increased costs [64,65,66,67].

Pancreatic enzyme replacement therapy (PERT) is cheap, mitigates against weight loss and improves quality of life. The most compelling argument for PERT is emerging evidence that it improves survival [46,58,68,69,70,71]. Given the failings of diagnostic tests for PEI and the evident benefits of PERT, it is recommended for all patients with pancreatic cancer by NICE in the United Kingdom [28].

A healthy pancreas is estimated to produce 900,000 United States Pharmacopeia (USP) of lipase in response to a meal. Sufficient absorption of fat can be maintained at around 10% of normal capacity, there is thus a need for around 90,000 USP per meal. Given that in the majority of pancreatic disease some function remains an appropriate starting dose in resected pancreatic cancer is 75,000 USP with a main meal and 25,000 with a snack [69], most effective when given across the course of, or just after, a meal rather than before [72]. Co-prescription of a proton pump inhibitor is often required after pancreatoduodenectomy as a failure to neutralise gastric acid leads to enzymes remaining inactive [73].

Pre-operative malnutrition is associated with significantly poorer post-operative outcomes for pancreatic resection, addressing this extends beyond just the prescribing of PERT [74,75]. The international Study Group on Pancreatic Surgery (ISGPS) released a position paper on nutritional support and therapy in pancreatic surgery, this emphasizes the importance of pre-operative assessment of nutritional status and recommends nutritional supplements in those who have, or are at risk of developing, moderate malnutrition [76]. Those with, or at risk of severe malnutrition may benefit from formal nutritional support with enteral or parenteral feeding [76]. Regular dietician input to asses response, compliance, diet, diabetic optimization, and the potential need for nutritional supplements is required [77].

Although outside the scope of this article to describe in detail, immunonutrition is worthy of note. Immunonutrition aims to influence the systemic immune system using nutritional supplements with immune modulating contents such as arginine, omega-3 fatty acid and RNA. This is not yet part of routine clinical practice, however, there is evidence that initiation in the pre-operative period could improve post-operative outcomes [78,79,80,81]. The most recent systematic review and meta-analysis of immunonutrition in pancreatic resection, concluded that immunonutrition reduces infectious complications (especially wound infection) and length of stay [82].

### 2.3. Benefits of and Access to Surgical Resection of Pancreatic Cancer in the Elderly

Benefits of resection do not diminish with increasing age [83,84]. Yet, many elderly patients are considered too frail for surgery and there is significant age-related disparity in access to surgery. Ageism is a major problem faced by elderly cancer patients; elderly patients without comorbidity are less likely to receive cancer therapy than younger patients with comorbidity. Advanced age, in the absence of comorbidity, is mistakenly considered a more significant barrier to surgery than comorbidity [85]. Elderly patients are less likely to undergo standard resection, less likely to undergo resection with concomitant venous resection and less likely to achieve negative margins [86,87,88]. An American population based study of over 45,000 patients with pancreatic adenocarcinoma observed that the rate of surgery decreased with increasing age: 21% of those under 50, 19% between the age of 50 and 70 and only 13% of those over 70 received surgery [87].

Concerns over risk and perceived lack of benefit of surgery are prohibitive and account for the discrepancies in care. However, a systematic review of surgery among elderly patients with pancreatic cancer demonstrated that over time, perioperative mortality has improved for elderly patients following pancreatic resection when compared to non-elderly patients, with mortality preceding the year 2000 being significantly higher in elderly patients, but similar from 2000 onwards [86]. Major surgical complications (post-operative pancreatic fistula, delayed gastric emptying, post pancreatectomy haemorrhage and surgical site infections) were similar between the elderly and the non-elderly; however, respiratory complications did occur more frequently in the elderly population. Prehabilitation can improve physical functioning and prevent deterioration among patients whilst waiting for cancer surgery [89,90,91,92,93,94]. The benefits of surgery are not diminished by age and therefore older patients with appropriate performance status should not be denied access based on chronological age alone. Treatment decisions for the elderly should be made in a multidisciplinary setting, should ideally include the use of a tool such as the comprehensive geriatric assessment and the input of a geriatrician to avoid discrepancies in treatment based on chronological age [95,96].

### 2.4. Prehabilitation

Resection is the only curative option for pancreatic cancer, however, the majority of patients with a new diagnosis are not suitable for operative management [97]. Baseline functional status influences receipt of curative resection, receipt of adjuvant chemotherapy, the rate and severity of post-operative complications and long term quality of life [89,98,99,100]. Functional status is often poor in patients with pancreatic cancer, this is contributed to by older age at diagnosis, pre-operative sarcopenia, malnutrition and obstructive jaundice [101]. Prehabilitation refers to any interventions, prior to definitive treatment that are aimed at improving patient health and lifestyle [102]. The concept centres around pre-operative conditioning to improve nutritional status and aerobic capacity. Studies of prehabilitation in other types of major surgery have suggested that these programmes can improve both access to definitive treatment, and post-operative outcomes [103] (See Table 3). A recent trial randomised patients to standard of care versus standard of care plus prehabilitation in patients having major abdominal surgery found that the prehabilitation cohort had improved aerobic capacity and a reduction in 30-day readmissions [104]. Unfortunately, systematic reviews and meta-analysis have determined that although prehabilitation programmes can potentially improve surgical outcomes, the evidence is weak, this is most likely do to the variation in prehabilitation regimens and study heterogeneity [102,103,105,106,107,108].

The majority of evidence for prehabilitation is limited to those undergoing colorectal resection, hepatic resection or major cardio-thoracic surgery, few studies look specifically at the impact of prehabilitation in pancreatic resection [109,110,111,112,113,114] (Table 1). Two studies have reported on the association between prehabilitation and post-operative outcomes in pancreatic cancer; Ausania et al. randomised patients to prehabilitation (*n* = 18) or standard of care (*n* = 22). Nakajima et al. compared a cohort of patients undergoing prehabilitation to historical patients. Neither study determined a significant difference in outcomes except for reduced rates of delayed gastric emptying in one and a shorter length of stay in the other. Several studies report an improvement in lean muscle mass prior to surgery and one reported improvement in quality of life in those undergoing prehabilitation [109,110,111,112,113,114]. Although showing promising results, the collective interpretation of these studies is difficult owing to poor standardisation of exercise and nutritional interventions and paucity of participants. Table 4: Pre-operative areas for potential gain.

## 3. Peri-Operative Pathways

### 3.1. Enhanced Recovery after Surgery

Enhanced recovery after surgery (ERAS) is a multimodal approach to a patient’s perioperative journey which aims to facilitate early return to the preoperative state [115]. Broadly, it facilitates sustained recovery and reduces complications. A secondary benefit is frequently a reduced length of stay.

The value of ERAS pathways in pancreatic surgery has been recommended on the basis of high level evidence in domains such as avoidance of hypothermia, use of wound catheters compared to epidural analgesia (EDA), use of somatostatin analogues to reduce CR-POPF [116]. protocols for thromboprophylaxis and antimicrobials and interventions for preoperative nutrition for patients with severe weight loss [36]. As patient reported outcomes (PROs) have become of particular importance in pancreatic cancer due to the elderly cohort of patients, a recent addition has been patient-centred PROs into the ERAS pathway [117,118].

The degree of compliance with ERAS pathways is strongly associated with clinical outcome [119]. With numerous components, intensive monitoring of the pathway with regular audit and a dedicated specialist nurse input improves compliance. An evaluation into the feasibility of an ERAS pathway after PD has demonstrated over 70% compliance achieved within a multicentre cohort study and was associated with a significant reduction of overall complications and length of stay [120].

ERAS after pancreatic surgery have been associated with a reduction in mild complications (Clavien Dindo grade I-II) significant improvements in overall morbidity and length of stay without any increase in readmission [121,122,123,124,125]. Effect on pancreatic specific complications is difficult to interpret as studies have variably included PD and distal pancreatectomy and ISGPS definitions have not been consistently reported for delayed gastric emptying (DGE) and postoperative pancreatic fistula (POPF) However, a lower incidence of DGE has been observed with no effect on POPF rate [122,123,125].

Implementation of ERAS pathways has been hindered due to the economic impact associated with the necessary resources required, namely a specialist nurse, audit and data collection and patient information booklets [126]. However, these costs, have been offset by the reduction in postoperative complications and subsequent hospital length of stay [127].

Though the short-term benefits are evident, long term benefits have also been suggested by one study where a relationship between increased compliance to the ERAS pathway and survival benefit has been found [128].

### 3.2. Reducing Complications from Surgery

Surgery alone, is a poor treatment for pancreatic cancer. Only with associated receipt of chemotherapy do patients gain a good chance for cure. One major barrier to patients receiving adjuvant therapy is the occurrence of post-operative complications. Thus, strategies to reduce these are attractive not only to improve perioperative outcomes but improve the delivery of adjuvant therapy. Post-operative pancreatic fistula (POPF) is the most frequent and severe complication after surgery [129]. Presently there is no widely accepted approach to reducing rates of clinically relevant POPF but a national study of early detection and minimally invasive treatment of POPF aims to determine whether the severity of POPF can be reduced [130].

An individual patient’s risk of POPF varies hugely. It is somewhat remarkable that POPF rates are not routinely adjusted to take this into account. Individual surgeons risk adjustment and CUSUM analysis is a way for surgeons to objectively assess their outcomes [131]. Such strategies could help inform surgeons of optimal techniques.

Strategies to improve outcomes after complex procedures such as pancreatoduodenectomy, evolve from a critical understanding of events and outcomes. Without critical analysis background noise, variations in practice and organisational differences can make this task insensitive. Determining the root causes of mortality after pancreatectomy demonstrates this well [129]. Only by conducting in depth analyses of processes and outcomes can surgeons begin to understand fundamental reasons for failure. Solutions can then be developed which are designed to overcome problems which are prevalent at the local level. Solutions are likely to vary from centre to centre dependent upon variation in practice and outcome.

A further key improvement is to determine benchmarks of optimal outcomes. This strategy seeks to reduce the effect of variation in practice between centres by focussing on common factors between centres and avoiding outlier cases. In pancreatic cancer, this has allowed teams to compare outcomes to those of their peers after surgery for resectable cancer or with associated venous resection [132,133].

Taking the concept of assimilated gains, the Dutch are leading the way with a nationwide implementation of best practices based upon critical analysis of pathways and suboptimal outcomes with the PACAP-1 trial which seeks to improve outcomes and overall survival [134]. Table 5: Summary of Peri-operative areas for gain

## 4. Post-Operative Pathways

Post-operative strategies should be designed to enhance functional recovery, maximise uptake of adjuvant chemotherapy and delivering appropriate surveillance. Care should be continuous and multidisciplinary, with continued nutritional consideration (as outlined in the pre-operative section). Functional decline and lack of chemotherapy uptake is more pronounced in elderly.

### 4.1. Adjuvant Therapy in Resectable Pancreatic Cancer

The benefit for adjuvant therapy is without question. Alone surgery achieves cure in less than 10% of patients [3,135]. Currently, a multimodal approach is the standard of care where 6 months of mFOLFIRINOX based on the results of PRODIGE-24 RCT for those with a sufficient performance status, or a combination of gemcitabine and capecitabine based on ESPAC-4 RCT [136,137]. Patients not sufficiently fit for combination therapy may still benefit from gemcitabine. The role of adjuvant therapy following neoadjuvant therapy and resection, however, is less clear. A recent multi-centre international study demonstrated that adjuvant chemotherapy following NAT, was only of benefit among patients with node positive disease [138].

Widespread variation in the use of adjuvant therapy is clear between and within countries. For example, studies demonstrate that 51% of patients receive adjuvant therapy in the USA, 54% in the Netherlands, 66% in Japan and 74% in Canada. Widespread variation within countries is also evident [139,140,141,142]. Within the Netherlands, rates of adjuvant therapy varied from 26 to 74% between health care providers. Sociodemographic variation explains some variation with deprived patients less likely to receive therapy [143]. Advanced age is a common factor associated with underuse of adjuvant therapy and will be considered separately below. However, such wide differences cannot be explained by demographic differences alone. Such data clearly points at systematic variation. Such variation is clearly undesirable and yet, despite the clear advantage of adjuvant therapy, there is little emphasis upon ensuring that patients chances of receiving therapy are optimized [144]. This low completion rate of the full therapeutic sequence may in part be explained by the reticence to initiate adjuvant chemotherapy following postoperative complications or poor performance status. However, ESPAC-3 has addressed this issue of time to initiate and optimal duration of chemotherapy [145]. Within this study, 68% of patients completed all six cycles of chemotherapy and for these patients, overall survival was significantly favoured. In those who did complete therapy, there was no difference in survival when comparing those who started earlier than eight weeks compared to those who started between 8 and 12 weeks, therefore time to initiation of chemotherapy was an important prognostic factor in favour of later treatment. Thus, this study concluded that completion of chemotherapy rather than early initiation was more important for survival.

Centralised cancer surgery is widely practiced. However, centralised chemotherapy is not standard practice. There is evidence that concentrating adjuvant therapy to a dedicated regional service increases the proportion of patients that receive therapy [146]. Overcoming nihilistic views and patients fear of therapy are important strategies as many patients choose not to pursue chemotherapy and clinicians not referring patients for chemotherapy is a mindset in great need of change [147].

A further variable that must be considered is not simply whether a patient receives adjuvant therapy but that efforts must focus upon an individualised approach where patients receive a regime that is as strong as can be tolerated for that individual. Incremental gains of multiagent therapy are seen over single agent gemcitabine [3,135,137]. Yet, there is very little data upon strategies to optimise not just the delivery of adjuvant therapy but also the regimen that is delivered. Some evidence supports centralised care to deliver more multiagent therapy [147].

Liquid biopsies are a novel way to diagnose cancer at an early stage, aid in prognostic evaluation [148], determine targets for therapy [149] and to evaluate cancer recurrence after treatment. The technique involves determining cancer DNA, vesicles and tumour cells in circulating blood [150,151]. Nomograms can be used to stratify patient risk for selection to treatment and may influence the choice of NAT, surgery or nature of adjuvant chemotherapy [152,153,154,155,156].

### 4.2. Benefits of and Access to Chemotherapy in the Elderly Population

After resection of pancreatic cancer, disease free survival (DFS) and disease specific survival (DSS) appear similar between elderly and younger patients, but overall survival (OS) is shorter, a possible reflection that elderly patients are less likely to receive adjuvant chemotherapy [86,157,158,159]. Elderly patients have just as much benefit from adjuvant therapy as younger patients and when chemotherapy use is stratified between young and older patients overall survival is the same [160]. The CONKO-001 trial had no upper age limit and specifically demonstrated that those over 65 show a similar improvement in OS and DFS as have numerous other studies evaluating adjuvant chemotherapy for pancreatic cancer [137,159,161,162,163]. This benefit is maintained when considering more aggressive regimens in the elderly such as FOLFIRINOX or neo-adjuvant chemotherapy [164,165,166]. Consequently, strategies to increase the use of chemotherapy in the elderly, such as centralisation of oncology services and measures to address frailty, yield major benefits [146,167]. The comprehensive geriatric assessment (CGA) is recommended by the Society for International Oncology in Geriatrics as a useful decision making tool for older people with malignancy [168]. There are several studies suggesting that the CGA can be useful in predicting functional decline, toxicity and overall survival [169,170] The CGA is lengthy and time consuming, a more practical approach, as recommended by the National Comprehensive Cancer Network, the European Organisation for Research and Treatment of Cancer and the International Society of Geriatric Oncology is to use a short frailty screening tool (such as the Fried score, the Clinical Frailty Scale or the Geriatric 8) to identify those who require a full screening [171,172,173,174,175,176,177].

Like surgery, the benefits of chemotherapy are not lessened by age and therefore older patients with appropriate performance status should not be denied access. Treatment decisions should be made in a multidisciplinary setting and include the use of a frailty screening tool and the input of a geriatrician to avoid discrepancies in treatment based on chronological age [95,96].

Since there are no published accepted standards for the proportion of patients that receive adjuvant therapy after pancreatic cancer surgery there is lack of clarity about what is acceptable practice. Thus, local audit and benchmarking one’s practice against peers is an essential step in the standardisation of practices. Quality improvement programs could further enhance delivery of adjuvant chemotherapy if they were to target those patients most at risk of not receiving therapy, i.e., to overcome barriers presented by age, frailty and good functional recovery after surgery.

### 4.3. Surveillance after Resection of Pancreatic Cancer

Surveillance after resection of other cancer types is routine and evidence based. The notion is simple, that early detection of recurrence is more likely to identify disease at an earlier asymptomatic phase when patients are more likely to have preserved performance status and ultimately more likely to receive treatment. Most guidelines, including those from the European Society for Medical Oncology (ESMO) and International Association of Pancreatology/European Pancreatic Club (IAP/EPC), do not recommend routine surveillance after pancreatic cancer resection due to the poor prognosis and limited treatment options available for recurrence [178,179]. The nature of surveillance protocols typically involve CT scans at 3 or 6 month intervals supplemented by CA19-9 analysis. The optimal interval and impact of surveillance programs remain to be seen. It may be that artificial intelligence may help refine and improve surveillance programs; there is currently much interest in AI as a tool to facilitate the diagnosis of breast cancer within screening programs.

In the setting of recurrent pancreatic cancer there are an increasing number of treatment strategies. Despite this, many patients fail to receive palliative therapy. However, an individualised approach to treating recurrent pancreatic cancer is logical. Around half of all patients recur with local only disease [180]. Whilst systemic therapy seeks to control occult metastatic disease local therapies in the form of high intensity radiotherapy or ablation can be delivered [181]. Given that many patients are elderly, frail or may have had poor experience with systemic therapy local therapy can be delivered with more less disruption and more satisfaction for the patient. Targeted therapy of oligometastatic disease also offers alternative options for a limited number of patients. Furthermore, surveillance is desired by patients and clinicians alike [182,183]. Table 6: Areas for Post-operative gain.

## 5. Novel Areas for Review

Though this body of work focusses upon areas where benefit can be clearly derived using existing evidence base there are areas with an emerging evidence base where benefit may be derived if practices are adopted.

Pancreatic surgery is associated with a high rate of cancer recurrence. Improving the staging pathways and novel approaches to treatment of the surgical margin are ways to improve outcomes.

Early cancer recurrence, even within ninety days of surgery, has to be considered a failure of staging [129]. The addition of routine Positron Emission Topography (PET) or Magnetic resonance imaging (MRI) scanning can help detect occult cancer. PET scanning, within a randomised trial, upstaged 20% of patients, preventing futile surgery [184]. However, it is important to note that the routine use of PET-CT is still debatable as it cause delays to resection and the potentially false positive results that may erroneously prevent surgery [185,186]. MRI can upstage 10–24% of patients with occult liver metastases where diffusion weight images can identify lesions under 5 mm, being more sensitive than computerised tomography (CT) or PET-CT [187,188,189]. These tests need to be considered carefully, however, as false positive results can be caused by common scenarios such as abscesses secondary to cholangitis and can delay treatment.

Failure of surgery to clear the margin is an Achilles heel of pancreatoduodenectomy. Local recurrence is very high after surgery, positive margins are associated with reduced survival and therefore it is necessary to consider how outcomes can be improved [190,191]. Neoadjuvant therapy (NAT) is associated with a higher rate of R0 resections, though it is important to consider results on an intention to treat basis [192,193]. There is much interest in NAT for resectable pancreatic cancer. Ongoing clinical trials will help determine the evidence base for this treatment. There are many retrospective cohort studies which are at risk of various bias including selection and survivor bias and until well conducted trial data is available it is not possible to make a clear statement [194]. Intraoperative frozen section has been used to identify positive pancreatic transection margins and the role of extending resections has been explored in numerous studies, but systematic review fails to observe a benefit to this practice [193]. Similarly, extending lymphadenectomy is not associated with greater survival but with morbidity [195,196]. The standard approach to pancreatoduodenectomy leaves perineural tissue around the superior mesenteric artery. It is this margin which is most frequently positive on histologic analysis. An artery first approach has a theoretical advantage that it can clear periadventitial tissues but clinical studies fail to demonstrate benefit in terms of improving R0 margin status [197,198]. Intraoperative radiotherapy has been long considered as an adjunct to improving margin status but no high-level evidence yet exists [199]. The addition of stereotactic ablative radiotherapy within a NAT program does not appear to improve R0 rates [200]. Irreversible electroporation has gained interest, predominantly surrounding control of locally advanced pancreatic cancer though the technology could be applied to improve margin control at resectable cancer. However, concerns over safety and a lack of high level evidence remain [201]. There is thus much work to be done in this area and focussing efforts on the problem of the surgical margin could reduce local recurrence.

Peritoneal metastases are less common than local recurrent or liver metastases but when they occur are associated with very poor outcomes. Positive intraoperative peritoneal cytology correlates with poor oncologic outcomes, in the absence of visible metastatic disease [202]. Among patients with cytology positive, or even macroscopic peritoneal disease, intraperitoneal chemotherapy can control ascites and some patients can go on to achieve surgical resection [203,204]. Such experience is limited to Japan. The remarkable outcomes indicate a potential role for intraperitoneal therapy, challenge current beliefs about pancreatic cancer biology and prognosis and merit a wider review.

Personalised medicine in pancreatic cancer care is most strongly associated with defining an individual’s cancer genetics. Large multinational, multicentre organisations/trials are ongoing such as precisionpanc (https://precisionpanc.org, accessed on 10 January 2021). There have, however, been many negative trials of genetic targets among patients with metastatic pancreatic cancer which include MEK inhibitors, IGFR inhibitors, mTOR inhibitors, TRK inhibitors, NOTCH inhibitors, TGF-β inhibitors, immunotherapy or vaccine therapy [205]. Established genetic targets in resectable pancreatic cancer are thus largely unclear; the most actionable mutation is BRCA1-2 which is identified in approximately 5% of patients [206]. Patients with these tumours are sensitive to platinum agents and thus will be treated by inclusion of oxaliplatin in therapy regimens [207]. This target has also been exploited by the PARP inhibitor Olaparib [208].

## 6. Conclusions

This review sought to elucidate key areas of variation, undertreatment and pathway changes where improvements can be realised with little effort. Novel therapeutic options will present themselves in the future, but it would be remiss of any team caring for this cohort of patients to inadequately utilise current evidence and implement optimal treatment pathways. There is a disconnect between funding for research to establish novel treatments far outstripping funding to implement best care at the level of the health care provider/organisation. It is essential that surgeons understand that surgery is just one part of a complex pathway and that they are ideally placed to act as change agents to optimise broader pathway improvements. Some changes can be clearly applied to the majority, if not all patients, such as PERT among those undergoing pancreatoduodenectomy. Other interventions can never be applied to all patients. There will always be some jaundiced patients with resectable cancer that undergo PBD or some patients who never receive adjuvant therapy. Locally, nationally or internationally accepted benchmarks are required to understand what is achievable and to help teams identify areas of poor performance. Collaborative multicentre, multinational studies are an essential part of assessing and improving patient care in the 21st century and teams are encouraged to develop and take part in these ventures. Through the aggregation of marginal gains, our patients can realise better outcomes and experience in the near future.

## Figures and Tables

**Table 1:** Absolute and relative indications for Percutaneous biliary drainage (PBD).

Indication for PDB		Comment
Absolute	Neoadjuvant therapy	
	Renal dysfunction	If mild, percutaneous external drainage and fluid replacement may permit early surgery with trans-sphincteric drainage to reduce the risk of pancreatitis
	Cholangitis with organ dysfunction	If mild, percutaneous external drainage and fluid replacement may permit early surgery with trans-sphincteric drainage to reduce the risk of pancreatitis
Relative	Bilirubin level	Exact cut off leve currently unclear, see text.
	Delay to surgery	Need to consider daily rate if increase in bilirubin as this may vary from patient to patient
	Malnutrition	Ensure exocrine insufficiency corrected and balance delays to surgery, cancer progression against severity of malnutrition
	Frailty	Frail patients are less likely to tolerate complications of PBD so a difficult discussion or choice often needs to be made. For some frail patients, direct to surgery may be there best chance to have surgery as delays or complications of PBD can exacerbate frailty

**Table 2:** Tips for optimising care in the event of PBD requirement.

Indication for PDB	Comment
Initial attempt at PBD	ERCP with self-expanding metal stent
Position of stent	The stent should be short and ideally not occlude the cystic duct origin as this can lead to cholecystitis which can delay surgery or chemotherapy
Periprocedural care	Antibiotics before and after the procedure Regular observations with escalation to medical team in event of abdominal pain and/or hypotension. Consideration of CT in event to exclude pancreatitis or perforation.
Options if initial PBD fails	Maximum of two attempts at ERCP. If unsuccessful for PTC with stent placement as a rescue option (referral to specialist centre may be optimal depending on local experience)
Definition of successful PBD	Biliary drainage is defined as successful if the serum bilirubin level decreased by 50% or more within 2 weeks after the procedure.

**Table 3:** Summary of prehabilitation studies for pancreatic resection.

	Year	Study Design	No.	Exercise Plan	Key Findings
Nakajima et al.	2019	Retrospective, cohort	76	30 days Unsupervised, self-reported exercise	Shorter length of stay in prehabilitation group (23 days vs. 30 days, *p* = 0.045)
Ausania et al.	2019	Randomised controlled trial	18	2 weeks supervised and unsupervised, aerobic exercise	Reduction in DGE in prehabilitation group (5.6% vs. 40.9%, *p* = 0.01)
Florez Bedoya et al.	2019	Prospective, cohort	23	15 weeks unsupervised, self-reported, aerobic and resistance exercise	No comment on clinical outcomes Evidence of prehabilitation increasing tumour vascularity.
Marker et al.	2018	Case series	3	12–16 weeks, supervised, physiotherapist reported, hour long, 3× a week	Underpowered
Parker et al	2018	Prospective, cohort	50	60 min per week unsupervised, self-reported aerobic and strengthening exercise	No comment on clinical outcomes Home based exercise programme feasible
Ngo-Huang et al	2019	Prospective, cohort	50	2 weeks, unsupervised, self-reported, hour long aerobic exercise	Prehabilitation associated with improved physical function (6MWT, STS, GS improved from baseline, *p* = 0.48, 0.03 & 0.08, respectively) and HRQOL (Improved with increased LPA (*p* = 0.01)

6MWT = 6 min walk test, STS = Sit to stand, GS = Gait speed, HRQOL = Health Related Quality of Life, LPA = Light physical activity.

**Table 4:** Pre-operative areas for potential gain.

Areas for Gain Pre-Operatively	Problem	Intervention	Gain
Avoidance of pre-operative biliary drainage	- PBD related complications are common - Avoidable delay to surgery - Missed window of opportunity for curative surgery	- Upfront surgery	- Avoid PBD related complications - Reduce postoperative complications - Increase number of patients undergoing curative surgery - Improve overall survival
Correction of PEI related malnutrition	- PEI is highly prevalent and related to weight loss, reduced QoL, reduced survival - Under-prescribing of PERT - Inadequate dosing - Malnourished at baseline - Sarcopenia	- PERT prescribing for all - Education of wider team - Patient education - Dietetic monitoring - Consider PPI - Consider supplements	- Improve quality of life - Reduce weight loss - Reduce complications - Improve survival
Prehabilitation	- Older age group - Sarcopenia - Too ‘frail’ for treatment - Deconditioning	- Consider who is ‘at risk’ - Physiotherapy involvement - More research	- Potential to reduce some complications - Reduce length of stay - Potential to improve access to treatment
Old age/frailty	- Nihilistic attitudes towards older age - Elderly not receiving same standard of care despite proven benefit	- MDT decision making with geriatrician if able - Use of assessment tools. Such as CGA - Co-morbidities rather than chronological age as deciding factor.	- Improved access to curative treatment options for the elderly.

PEI = Pancreatic exocrine insufficiency, PBD = Pre-operative biliary drainage, QoL = Quality of life, PERT = Pancreatic enzyme replacement therapy, PPI = Proton pump inhibitor, CGA = Comprehensive geriatric assessment.

**Table 5:** Summary of Peri-operative areas for gain.

Areas for Gain Peri-Operatively	Problem	Intervention	Gain
ERAS	- Protocol compliance - Standardisation of practice	- Standardised practices based upon best evidence - Routine audit	- Reduced complications - Reduced Hospital Costs - Reduced LoS
Reducing complications	- QI programs - Benchmarking - Root cause analysis	- Improved outcomes with POPF though systematic changes and better understanding of risk - Benchmarking and root cause analysis demonstrate where local outcomes fall below expected outcomes or those of peers	- Reduced complications and reduced harm from complications - Improved access to adjuvant chemotherapy - More rapid functional recovery after surgery
Correction of PEI related malnutrition	- See Table 2	- Junior Dr education - Patient education - PERT prescribing check - Dietetic review - See Table 4	- See Table 4

ERAS = Enhanced Recovery after Surgery, QI = Quality Improvement, POPF = Post-Operative Pancreatic Fistula, LoS = Length of Stay, PERT = Pancreatic Exocrine Replacement Therapy.

**Table 6:** Areas for Post-operative gain.

Areas for Gain Post-Operatively	Problem	Intervention	Gain
Adjuvant chemotherapy	- Nihilism and under-utilisation of adjuvant chemotherapy	- Establish standards of practice and benchmarks for the proportion of patients that receive adjuvant therapy - QI programs to address frailty, advanced age and recovery after surgery	- Improved uptake of adjuvant therapy - Improved survival - Standardised practices
Surveillance	- Lack of standardised practice - Nihilism	- Implement surveillance programs	- Improved detection of early recurrence - Improved treatment rates - Improved duration of survival
Nutrition	- See Table 4	- Education of local physicians and those in palliative care - See Table 4	- See Table 4

QI = Quality improvement.

## Data Availability

Not applicable.
